# The complex relationship between estrogen and migraines: a scoping review

**DOI:** 10.1186/s13643-021-01618-4

**Published:** 2021-03-10

**Authors:** Nihaal Reddy, Miraj N. Desai, Anna Schoenbrunner, Steven Schneeberger, Jeffrey E. Janis

**Affiliations:** 1 The Ohio State University College of Medicine, Columbus, OH USA; 2 Department of Plastic and Reconstructive Surgery, The Ohio State University Wexner Medical Center, 915 Olentangy River Rd, Columbus, OH 43212 USA

**Keywords:** Migraine, Estrogen, Menstrual-related migraines, Headache, Hormones, Hormonally mediated migraines, Migraine surgery, Migraine decompression, Female migraines

## Abstract

**Background:**

Migraines are a chronic disease for millions worldwide and have been hypothesized to be hormonally mediated due to their higher prevalence in females and menstrual associations. Estrogen has been commonly implicated in migraine pathogenesis, yet its exact role in the pathophysiology of migraines has yet to be fully understood.

**Method:**

We conducted a scoping review of the literature regarding estrogen’s role in migraine pathogenesis and included 19 studies out of an initial 202 in the final review. Two independent reviewers screened and extracted data from included studies based on predetermined inclusions and exclusion criteria.

**Results:**

The estrogen withdrawal hypothesis, discussed by 12 of the reviewed studies, is the most discussed theory about estrogen’s role in migraine physiology and describes the association of migraine onset with natural declines in estrogen levels, particularly when estrogen levels fall below 45–50 pg/mL after an extended period of priming. Additional findings suggest that women with a history of migraine have an increased sensitivity to physiologic fluctuations in estradiol levels. Several studies suggest that migraines are associated with menstruation.

**Conclusion:**

It appears that estrogen is very likely to play a key role in migraine pathogenesis, but seems to affect patients in different ways depending on their past medical history, age, and use of hormonal therapy. Further research is warranted to isolate the effects of estrogen in each unique patient population, and we believe that studies comparing menstruating women to postmenopausal women could help shed light in this area.

## Background

Worldwide, millions of patients suffer from migraine headaches. The prevalence of migraines is significantly higher in women (18% vs. 6%) [1], who are also more likely to experience more intense and longer lasting migraines compared to men [2]. Despite their prevalence, their definitive pathogenesis is still an active area of research. It has been noted that puberty and menopause are the time periods most associated with migraines in women; up to 70% of female migraineurs notice menstrual association of their headaches [3]. Menstrual-related migraines are reported to be longer, more painful, and more resistant to treatment than non-menstrual-related migraines [4].

As migraines are more frequent among females, a variety of hormones have been implicated in their pathogenesis; specifically, prior research has repeatedly shown evidence linking estrogen to migraine headaches [5]. Pringsheim highlighted this association when they found that the prevalence of migraines in male-to-female transgender individuals taking estrogen therapy was similar to the prevalence of migraines in females, and far higher than that in males [6]. Although numerous studies have suggested that estrogen plays a leading role in migraine pathogenesis, its specific role has yet to be fully understood. The purpose of this review is to investigate the specific roles of estrogen in the pathophysiology of migraine headaches to give providers and patients a better understanding of migraine pathology, treatment options, and areas of potential future research.

## Methods

### Inclusion criteria

In our literature search, our primary focus was to find studies that investigated the role of estrogen in the pathogenesis of migraine. We kept our search broad and utilized the inclusion and exclusion criteria below to narrow down search results in accordance with our predetermined PICO framework.

**Table:** 

Inclusion criteria	Exclusion criteria
*English language*	*Medical management of migraines*
*Females (>18 years old) with chronic or menstrual migraine*	*Focus on non-estrogen hormones and neurotransmitters*
*Impact of estrogen on migraine*	*Non-clinical research (e.g., basic science, animal-based, translational)*
*All study designs*	*Systematic reviews, meta-analysis, and literature reviews, letters to editor*

### Information sources and search strategy

A scoping review was undertaken to identify studies pertaining to estrogen and migraine headaches. PupMed and EMBASE were searched for articles published in the English language using keywords and the respective MESH and PICO search engines. Our data collection and extraction is highlighted below in Table 1 and the PRISMA 2009 Flow Diagram.

**Table 1:** GRADE Quality of Evidence Assessment

Study	Study Design	Risk of Bias	Imprecision	Inconsistency	Indirectness	Publication Bias	Comments	Quality
Cachrimanidou 1993 [7]	RCT	Low	Not serious	Not serious	Not serious	Not serious	N/A	High
Brandes 2006 [5], Misakian 2003 [8]	Cross sectional	Low	Not serious	Not serious	Not serious	Not serious	Both studies used data from Women's Health Study	High
Amir 2005 [9]	Cross sectional	Low	Serious	Serious	Not serious	Not serious	Potential confounding from participants being treated with IVF. Inconsistency with other studies due to conclusion that GnRH therapy increased risk of migraine. Potential imprecision due to to small sample size.	Moderate
Calhoun 2008 [4]	Prospective cohort	Some	Serious	Serious	Not serious	Not serious	Potential confounding from participants having a history of medically intractable menstrual migraine. Inconsistency with other studies and imprecision due to 36% of participants in treatment group having no statistically significant change in outcome and being excluded from treatment group in final analysis.	Moderate
de Lignieres 1986 [10]	Double blind placebo-controlled crossover	Some	Serious	Not serious	Not serious	Not serious	Older age of participants and small sample size	Moderate
Facchinetti 2002 [11], Martin 2003 [12]	RCT	Some	Serious	Not serious	Not serious	Not serious	Older age of participants and small sample size	Moderate
MacGregor 2006 [13]	Double blind placebo-controlled crossover	Some	Serious	Not serious	Not serious	Not serious	Nonspecific participant selection criteria. Imprescision due to wide confidence intervals and small sample size	Moderate
Calhoun 2004 [14]	Open-label clinical trial	Some	Serious	Not serious	Not serious	Not serious	Lack of control and blinding. Imprecision due to small sample size and lack of control to compare results	Low
Johannes 1995 [15]	Self-reported 4-month diary	High	Serious	Not serious	Not serious	Not serious	Imprecision due to small sample size and lack of control to compare results	Low
Lichten 1995 [16]	Crossover	Some	Very serious	Not serious	Serious	Not serious	Potential confounding from participants having a history of medically intractable menstrual migraine. Indirectness present due to results not discussing the difference in response to treatment between participants, which is what the study design was for. Additionally, only women women that initially responded to treatment were progressed in the study. Considerable imprecision from small sample size and lack of consistent statistical analysis.	Low
Mattsson 2003 [17]	Survey	High	Very serious	Not serious	Not serious	Not serious	Increased risk of bias and imprecision due to self-reporting study design	Low
Pringsheim 2004 [6]	Survey	High	Very serious	Not serious	Not serious	Not serious	Increased risk of bias and imprecision due to self-reporting study design and lack of control group.	Low
Stewart 2000 [18]	Self-reported 98 day diary	High	Very serious	Not serious	Not serious	Not serious	Increased risk of bias and imprecision due to self-reporting study design and lack of control group.	Low
Lichten 1996 [19]	Open-label clinical trial	High	Very serious	Not serious	Not serious	Not serious	Lack of control group and single time administration. Imprecision due to small sample size and lack of control to compare results	Very low
Marcus 1999 [20], Murray 1997 [21]	Prospective cohort	High	Very serious	Serious	Not serious	Not serious	Very small sample sizes and potential confounding variables of pregnancy and / or hormone replacement therapy. Inconsistency due to conclusion that GnRH therapy decreased headache index. Imprecision due to small sample size and lack of control to compare results	Very low
Lichten 1996 [19]	Open-label clinical trial	High	Very serious	Not serious	Serious	Not serious	Small sample size and potential confounding from HRT use. Indirectness due to conclusions focusing on specific estrogen serum levels that precipitate migraine while study data focused on effect of timing in relation to withdrawal of estrogen on precipitation of migraine. Imprecision due to small sample size and lack of consistent statistical analysis	Very low
Somerville 1972 [22]	Crossover (case series)	High	Very serious	Not serious	Not serious	Serious	Small sample size and lack of statistical analysis. Potential risk of publication bias due to positive study results and being one of the first studies published on the topic. However this study is considered a landmark study in the field of hormonal migraines.	Very low

### MESH search engine construction

Our MESH search was constructed as the following: (“migraine disorders”[MeSH Terms] OR (“migraine”[All Fields] AND “disorders”[All Fields]} OR “migraine disorders [All Fields] OR “migraine’ [All Fields] AND (“oestrogen”[All Fields] OR “estrogens” [Pharmacological Action] “estrogens” [MeSH Terms] OR “estrogen” [All Fields] OR “estrogen” [All Fields] AND chronic [All Fields].

### PICO search engine construction

The PICO engine of EMBASE was utilized in order to retrieve articles. Our search terms included [transformed migraine OR migraine], [estrogen], [chronic OR persistent OR recurrent] and were kept purposefully broad in order to return as many relevant papers as possible. Suggested synonyms for each term were selected to be included in the search. Papers were then screened in accordance with our PICO framework and inclusion/exclusion criteria to narrow down the search results.

**Table:** 

**P**opulation	• Menstruating women with menstrual or chronic migraine • Postmenopausal women with chronic migraine • Pregnant women with history of migraines • Transgender men and women with history of migraine
**I**ntervention	• Estradiol (injection, percutaneous, etc.) • Hormone replacement therapy • GnRH agonists • Oral contraceptives
**C**omparison	• Women with non-hormonal chronic migraine • Women with non-menstrual intermittent migraine
**O**utcomes	• Migraine frequency • Migraine severity • Headache index* • Delay of menstrual migraine • Onset of migraine relative to menstrual cycle

*Headache index is a metric for headache frequency, severity, and duration

### PUBMED supplementary search

General searches were also conducted on PUBMED using the keywords *migraine, estrogen, sex hormones, menstrual migraine, menstrual-associated migraine, menstrual-related migraine, migraine and estrogens, migraine and sex hormones, estrogen and headaches,* and *sex hormones and headaches.* Bibliographies of relevant articles were also examined in order to identify other potentially pertinent articles.

### Data collection and analysis

#### Data extraction and management

Relevant articles were uploaded and maintained in the program *Covidence.* Data extraction was performed by two independent reviewers. A premade summary of findings table was filled out by one reviewer which included the type of study, number of participants and their characteristics, the study interventions and outcomes, and conclusions drawn by each study with a summary of statistical analysis. In studies with multiple outcomes, we chose only to extract outcomes that were in accordance with our inclusion and exclusion criteria. A second reviewer then assessed the extracted data for accuracy. In cases of disagreement between both reviewers, a third reviewer made the final decision.

#### Quality of evidence

To evaluate quality of evidence, we utilized the Grading of Recommendations, Assessment, Development and Evaluations (GRADE) framework and assessed each study for risk of bias, imprecision, inconsistency, indirectness, and publication bias. Based on these five criteria, studies were categorized as very low, low, moderate, and high GRADE. This process was started by one reviewer and verified by a second reviewer. Any disagreements were settled by a third reviewer. Quality of evidence results can be seen in Table 1 below.

## Results

### Selection of studies

**Fig. 1:**
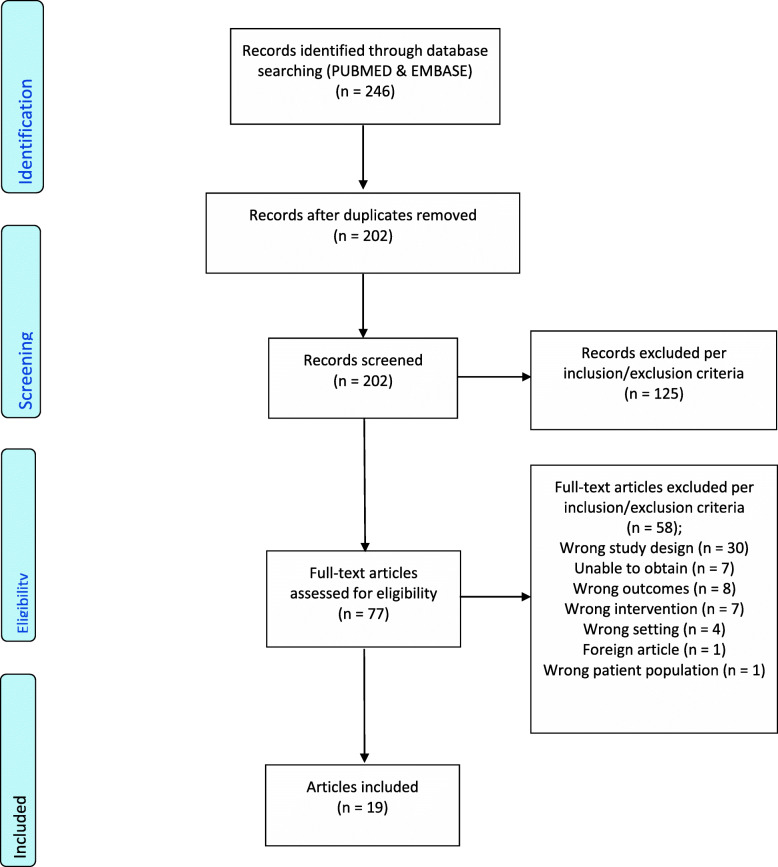
PRISMA 2009 Flow Diagram; Table 1, summary of articles

A total of 246 studies were imported for initial screening. Abstracts were initially screened by two blinded reviewers. Each reviewer screened each abstract, and studies that were agreed on by both reviewers advanced to full-text screening. Seventy-seven full-text studies were reviewed by the two blinded reviewers. Each reviewer screened each full-text article and studies deemed appropriate by both reviewers were included in the final review. After excluding any nonclinical studies as well as studies that did not directly study estrogen, 19 studies were included in the final review. This process is summarized in the PRISMA flow diagram in Fig. 1. A summary of findings can be seen in Table 2.

**Table 2:** Summary of Findings

Article	# of particpants	Patient characteristics	Intervention	Outcome/conclusions	Study design	GRADE
Amir, 2005 [9]	98	Menstruating women undergoing IVF and being treated with GnRH agonist (mean age 33.5)	Use of GnRH agonist	Use of GnRH agonist reduced estradiol levels to nearly undetectable and was associated with increased risk of migraine by 28.6% (95% CI 19.7-37.5%)	Cross sectional (Soroka medical center in Israel)	Moderate
Brandes, 2006 [5]	18221	Postmenopausal	Estrogen dosing in HRT; low (< 0.3 mg/day), intermediate (0.625 mg/day), high (> 0.9 mg/day)	Intermediate dose estrogen in HRT had a significantly lower risk of migraine occurrence (OR 1.28, 95% CI 1.10-1.48, p=0.001) than low (OR 2.00, 95% CI 1.51-2.65, p=0.001) or high (OR 1.72, 95% CI 1.39-2.13, p=0.002) dose compared to general population	Cross sectional (Part of Women’s Health Study)	High
Cachrimanidou, 1993 [7]	300	Menstruating women age 18-39 that attended a family planning clinic to request oral contraceptives	Study group took combined OCP with 30 ug ethinyl estradiol (low dose) with a 9 weeks on, 1 week off regimen. Control group took same OCP with a 3 weeks on, 1 week off traditional regimen	Women on the 9 weeks on, 1 week off regimen reported a 9.7% incidence of migraine complaints compared to 17.3% in women on the traditional 3 weeks on, 1 week off regimen (p < 0.01). Concluded that estrogen replacement delayed estrogen withdrawal menstrual migraine	RCT (multicenter study of three clinics in Sweden)	High
Calhoun, 2004 [14]	20	Menstruating women with history of menstrual related migraine (< 14 days of headache per month)	20 ug ethinyl estradiol on days 1-21 of menstrual cycle (percutaneous and oral) with 0.9 mg conjugated equine estrogens on days 22-28 (the placebo week for OCPs)	Estrogen replacement during placebo week of OCP treatment significantly reduced migraines experienced per month by 76%, from 7.6 headache days per month to 1.6 after treatment. Concluded that estrogen replacement prevented estrogen withdrawal migraine	Open-label clinical trial	Low
Calhoun, 2008 [4]	229	Menstruating women that were referred to an academic center for intractable menstrual migraines (mean age 35.6)	Ethinyl estradiol-containing oral contraceptive dosed to prevent premenstrual ethinyl estradiol decline by more than 10 mcg	In women that experienced resolution of menstrual migraine after treatment, there was a 55.8% reduction in the number of headache days per month (p < 0.001) compared to the 36% of women that continued to experience persistent menstrual migraine with no reduction in frequency	Prospective cohort (single-center uncontrolled study)	Moderate
de Lignieres, 1986 [10]	20	Menstruating women that experienced menstrual migraines in last 12 menstrual cycles (mean age 42.5)	Percutaneous administration of 1.5 mg estradiol in 2.5 g gel 48 hours prior to expected migraine	31% of women treated experienced a menstrual migraine after treatment compared to 96% of women in placebo group (*p* < 0.01). Concluded that physiologic withdrawal of estrogen precipitates menstrual migraine and that treatment with supplemental estrogen can prevent withdrawal migraine.	Double blind placebo-controlled crossover (single-center study in UK)	Moderate
Facchinetti, 2002 [11]	38	Postmenopausal women with history of migraine (mean age 51.1)	HRT with estradiol hemi- hydrate 1 mg/day plus norethisterone 0.5 mg/day for 28 days, in a continuous combined scheme; oral conjugated estrogens 0.625 mg/day for 28 days plus medroxyprogesterone acetate 10 mg/day in the last 14 days, in a sequential continuous scheme; and estradiol valerate 2 mg/day for 21 days plus cyproterone acetate 1 mg/day from day 12 to 21 in a sequential cyclical scheme	All 3 HRT treatments significantly increased migraine attack frequency (2.2 days per month vs 3.8, *p* < 0.001), (3.4 days per month vs 4.9, p < 0.001), (3.4 days per month vs 5.6, *p* < 0.001) over the course of 6 months. Patients also reported increased severity of headache after starting therapy	RCT (single-center study in Italy)	Moderate
Johannes, 1995 [15]	74	Menstruating women age 22–29 with history of menstrual migraine	Relation of migraine timing to menstrual cycle	There was a significantly higher incidence of migraine during the first 3 days of menses compared to remainder of menstrual cycle (OR 1.66, 95% CI 1.21–2.26)	Self-reported 4-month diary	Low
Lichten, 1995 [16]	29	Menstruating women with recurrent, medically unresponsive, menstrual migraine	Treatment for 2 months, first with placebo, then depo-leuprolide acetate 3.75 mg. Those who remained migraine free at 2 months continued therapy for 12 months with added transdermal estradiol	17/29 women remained migraine free at 2 months, 14/29 remained migraine free at one year. The initial 17 women had an average 50% improvement in headache index while taking therapy compared to placebo. Concluded that ovarian hormones are responsible for migraine pathogenesis and that reducing physiologic fluctuations in estrogen can reduce migraine incidence	Crossover	Low
Lichten, 1996 [19]	28	Postmenopausal women with history of severe menstrual migraine prior to menopause and taking HRT	One-time dose of 5 mg depo-estradiol cypionate intramuscular injection	All participants experienced a severe migraine on day 18 ± 4 of the study with average serum estradiol level on day of migraine between 45 and 50 pg/mL. No control group participants experienced a migraine during the course of the study. Concluded that there may be a genetic component to migraine pathogenesis and that estradiol levels falling below 50 pg/mL after a period of priming with higher estradiol levels can be a trigger for migraine	Open-label clinical trial	Very low
MacGregor, 2006 [13]	35	Menstruating women with menstrual migraine in last 3 menstrual cycles	Daily administration of percutaneous estradiol gel (1.5 mg estradiol) from 6 days prior to onset of menses until day 2 of menses	22% reduction in migraine incidence while using percutaneous estradiol gel (RR 0.78, 95% CI 0.62–0.99, *p* = 0.04) with 40% increase in migraine occurrence in the 5 days after discontinuing the intervention (RR 1.40, 95% CI 1.03–1.92, *p* = 0.03). Concluded that estrogen replacement can delay onset of estrogen withdrawal menstrual migraine	Double blind placebo-controlled crossover	Moderate
Marcus, 1999 [20]	49	Pregnant women with history of chronic headache (mean age 29.4, mean headache duration 9.1 years)	History of chronic headache	Women with history of chronic headache reported a 30% decrease in headache frequency during the 2nd and 3rd trimesters of pregnancy. This was not a statistically significant difference compared to women with no history of chronic headache. Concluded that women with chronic headache in the 1st trimseter of pregnancy would likely continue to have headaches through pregnancy and postpartum	Prospective cohort	Very low
Martin, 2003 [12]	21	Menstruating women with history of menstrual migriane (mean age 39)	Both groups treated with GnRH agonist to simulate medical oophorectomy, with one group getting estrogen add-back therapy to maintain estradiol at > 50 pg/mL (prior studied threshold for triggering menstrual migraine)	In the group that recieved estrogen add-back therapy to maintain estradiol levels at > 50 pg/mL, the rise in estradiol on days 1 and 2 of the study increased headache index by 45% compared to day 6. During the overall study duration, estrogen add-back therapy reduced headache index by 33.7% compared to the control group (95% CI, 3.0–64.4%). Concluded that small rises in estrogen can precipitate migraine in some patients, and preventing estrogen withdrawal can prevent migraine in some patients. Patients with history of menstrual migraine are very sensitive to changes in estrogen levels	RCT	Moderate
Mattsson, 2003 [17]	728	Women aged 40–74 with history of menstrual migraine	Relation of migraine timing to menstrual cycle	75% of women reported that their menstrual migraines occurred within day -2 to +3 of menstrual cycle	Survey	Low
Misakian 2003 [8]	17107	Postmenopausal	Use of HRT	HRT use significantly increased risk of experiencing a migraine in postmenopausal women (13% vs 9%, *p* < 0.001)	Cross sectional (Part of Women’s Health Study)	High
Murray, 1997 [21]	5	Menstruating women with repetitive severe migraines limited only to the perimenstrual period	3.75 mg IM depot-leuprolide acetate monthly injections for 10 months with 0.1 mg daily transdermal ethinyl estradiol added from month 5 onwards	74% decrease in headache index when being treated with only GnRH analog and 80% decrease after estrogen was added back. Concluded that stabilization of fluctuations in estrogen decreases incidence of menstrual migraine	Prospective cohort	Very low
Pringsheim, 2004 [6]	50	Male-to-female transgenders taking antiandrogen and estrogen therapy with history of migraine	Male-to-female transgender vs. general population	Prevalence of migraines in male-to-female transgenders (26%) was significantly higher than genetic males (7.5%) in the population (p < 0.05), but was not significantly different from prevalence of migraines in genetic females (25%) in the population	Survey	Low
Somerville, 1972 [22]	6	Menstruating women with history of menstrual migraine	Administration of estradiol valerate (10 mg) to maintain high plasma estradiol level during premenstrual and menstrual phases	Migraines attacks were delayed by 3 to 9 days (relative to usual timing during each patient's menstrual cycle) when treated with estradiol valerate, suggesting that migraines are precipitated by a fall in estradiol levels, particularly when they fall below 45-50 pg/mL during the perimenstrual period	Crossover (case series)	Very low
Stewart, 2000 [18]	81	Menstruating women age 18–55 with history of menstrual migraines	Relation of migraine timing to menstrual cycle	There was an increased incidence of migraine perimenstrually. Days 0 and 1 had OR 2.04 (95% CI 1.49–2.81). Days -1 and -2 had OR 1.80, 95% CI 1.40–2.30)	Self-reported 98 day diary	Low

The 19 studies included in our review consisted of 3 randomized controlled trials (RCT), 3 cross-sectional studies, 3 prospective cohort studies, 2 open-label clinical trials, 4 crossover studies (2 of which were double-blind placebo-controlled), and 4 survey/diary-based studies consisting of 37,139 total patients. The patients in these studies included 1646 menstruating women, 35,394 postmenopausal women, 50 male-to-female transgender women, and 49 pregnant women. Further details of patient characteristics are summarized in Table 1. Of the 19 studies, 12 studies investigated the effect of estrogen withdrawal on migraine occurrence and concluded that the withdrawal of estrogen during menses is a key factor in the precipitation of migraines, with the majority of these studies focusing on menstrual migraines in premenopausal women [4, 7, 9, 10, 12–15, 17–19, 22]. In particular, two studies found that priming with estrogen followed by a drop in serum estradiol levels below 45–50 pg/mL increased the risk of migraine precipitation [19, 22].

Three studies assessed the effects of hormone replacement therapy (HRT) on migraine in postmenopausal women with results varying by estradiol dosing, but generally concluding that estrogen replacement increased the incidence of migraine [8, 11, 23]. Three other studies found that women with a history of migraine had an increased sensitivity to physiologic fluctuations in estradiol levels [12, 16, 21]. Lastly, four survey/diary-based studies suggested that migraines most often occurred perimenstrually and were more common in male-to-female transgender patients on HRT when compared to the general population [6, 15, 17, 18]. These results suggest that estrogen is a major hormone implicated in migraine pathogenesis and that physiologic withdrawal of estrogen during menses likely plays a role in this condition. However, one study followed pregnant women with a history of migraine and found that migraine frequency did not change significantly as pregnancy progressed [20].

### The initial link between estrogen and migraines: estrogen withdrawal hypothesis

Estrogen’s association to migraines was first demonstrated by Somerville in 1972, providing for the first time an explanation for menstrual migraines [22]. Somerville found that he was able to delay menstrual migraine attacks by up to 9 days by treating participants with supplemental estrogen. He noted that migraines were particularly triggered when estradiol levels fell below 45–50 pg/mL during the perimenstrual period. Somerville concluded that estrogen plays a large role in the precipitation of menstrual migraines and that estrogen withdrawal during menses is the primary trigger. Although these claims inspired decades of research on the topic, Somerville’s study only consisted of 6 subjects and was prone to confounding bias due to some participants being close to the age of menopause.

The phenomenon of estrogen withdrawal was later studied by Lichten in 1996 in 28 postmenopausal women taking HRT and with a history of severe menstrual migraines prior to menopause [19]. Lichten administered a one-time intramuscular injection of 5 mg depo-estradiol cypionate and serially tracked serum estradiol levels until all participants experienced a migraine. He found that all participants in the experimental group experienced a migraine 18 ± 4 days after administration of the injection and that the average serum estradiol level at the time of migraine was between 45 and 50 pg/mL, as Somerville had found in his study. However, no participants in the control group (no prior history of migraine) experienced a migraine during the course of the study. Lichten concluded that is likely a genetic component in migraine pathogenesis and that a drop in estradiol levels to below 50 pg/mL after a period of priming with higher levels can be a trigger for migraine. Like the Somerville study, we assigned this study a very low GRADE score because of the limited sample size and confounding effects of ongoing HRT by participants.

### Estrogen withdrawal hypothesis—supporting studies

In support of Somerville findings, we found six studies in our review that investigated the role of estrogen withdrawal in the precipitation of menstrual migraine. In 1986, de Lignieres conducted a double-blind placebo-controlled crossover study based on Somerville’s initial project and also concluded that physiologic withdrawal of estrogen precipitates migraine and supplementing estrogen can prevent migraine [10]. De Lignieres administered 1.5 mg of percutaneous estradiol 48 h prior to expected migraine onset in menstruating women with a history of consistent menstrual migraine in their last 12 menstrual cycles and found that only 31% of participants experienced a menstrual migraine after treatment compared to women in the placebo group. While this was consistent with Somerville’s work and improved on the study design, it was limited by the small sample size of 20 women and advanced age of participants (average age 42.5).

In 1993, Cachrimanidou’s multicenter RCT of 300 patients at family planning clinics in Sweden found that changing the oral contraceptive (OCP) dosing regimen from a traditional 3 weeks on, 1 week off schedule to a 9 weeks on, 1 week off schedule reduced the frequency of migraine from 17.3 to 9.7% (*p* < 0.01) [7]. We considered this study to be high GRADE due to the large sample size and RCT study design. Cachrimanidou’s study was bolstered in 2006 when MacGregor found that daily administration of percutaneous estradiol gel (1.5 mg) from 6 days prior to menses until day 2 of menses resulted in a 22% reduction of migraine frequency (RR 0.78; 95% CI, 0.62–0.99, *p* = 0.04) [13]. However, MacGregor also found that there was a 40% increase in migraine frequency 5 days after discontinuing the intervention (RR 1.40; 95% CI, 1.03–1.92, *p* = 0.03). Cachrimanidou’s and MacGregor’s findings indicated that preventing estrogen withdrawal prior to menses can delay estrogen withdrawal migraine.

Calhoun conducted two studies that also supported the role of estrogen withdrawal in migraine pathogenesis [14]. In 2004, Calhoun followed 20 women with a history of menstrual migraine and showed that estrogen replacement with 0.9 mg conjugated equine estrogens during the placebo week of OCP dosing significantly reduced migraines experienced per month by 76% (7.6 vs. 1.6 headache days per month) offering evidence that preventing the withdrawal of estrogen can reduce migraine incidence [14]. Calhoun’s group continued their efforts in 2008 investigating a prospective cohort of 229 menstruating women with intractable menstrual migraines showed that using ethinyl estradiol-containing oral contraceptives to prevent premenstrual estradiol decline by more 10 mcg reduced migraine days per month by 55.8% (*p* < 0.001) for women that experienced a resolution of their menstrual migraine [4]. However, 36% of women in the study continued to experience persistent menstrual migraines with no reduction in frequency.

In 2003, Martin conducted a RCT of 21 menstruating women that were treated with GnRH agonist therapy to simulate medical oophorectomy [12]. The experimental group was given estrogen add-back therapy to maintain serum estradiol at > 50 pg/mL (reported in prior studies as the threshold for triggering menstrual migraine) and was found to have 33.7% reduction in headache index (95% CI, 3.0–64.4%). However, Martin also found that participants in the experimental group had a 45% increase in headache index during days 1 and 2 of the study, which he attributed to the rise in serum estradiol immediately after estrogen add-back therapy was initiated. Martin concluded that both estrogen withdrawal and rise can precipitate migraines and that patients with a history of menstrual migraine are very sensitive to changes in serum estradiol levels.

### Studies investigating fluctuations of estrogen on migraines

Martin was not the first author to suggest that fluctuations in estrogen may contribute to migraine pathogenesis. In 1995, Lichten’s group investigated 29 menstruating women with medically intractable menstrual migraines who were treated for two months with 3.75 mg depo-leuprolide acetate to effectively induce medical menopause [16]. Of the 29 women treated, 17 remained migraine-free at 2 months and reported an average 50% improvement in headache index. These 17 women were then treated for 10 more months with transdermal estradiol add-back therapy to maintain estradiol levels at a steady value. At the 1-year mark, 14 women remained migraine-free. This study was able to conclude that ovarian hormones play a key role in migraine pathogenesis and that reducing physiologic fluctuations in estrogen can help reduce the incidence of migraine. In a nearly identical study in 1997, Murray treated five menstruating women with intractable menstrual migraines with the same dose of depo-leuprolide acetate for 10 months with 0.1 mg daily transdermal ethinyl estradiol added back from month 5 onwards [21]. Murray found that participants reported a 74% decrease in headache index after being treated with only GnRH analog therapy, and this increased to 80% after estrogen add-back therapy at 5 months. Murray also concluded that minimizing fluctuations in estrogen can reduce migraine incidence.

However, Amir’s study in 2005 contradicted Murray’s initial finding that treatment with only GnRH analog therapy reduced headache index (a metric for headache frequency, severity, and duration) [9]. Amir studied 98 women undergoing in vitro fertilization with GnRH agonists and found that use of GnRH agonists to reduce serum estradiol levels was associated with a 28.6% increased incidence of migraine (95% CI, 19.7–37.5%). In our assessment of study quality, we gave Murray’s findings a very low GRADE score due to the limited sample size and lack of statistical analysis. On the other hand, Amir’s study was given a moderate GRADE score because of the study design, sample size, and narrow confidence interval. Overall, these studies suggest that physiologic fluctuations in estrogen may play a role in migraine pathogenesis, but complete elimination of estrogen may precipitate migraines.

### Survey studies investigating menstrual-related migraines

Four survey-based studies were included in our review, and all were found to support Somerville’s estrogen withdrawal theory. However, these were graded to be low-tier evidence due to possible reporting biases. Mattsson’s study surveyed 728 women and concluded that 75% of these women reported migraines occurring within –2 to +3 days of menstruation [17]. However, this study surveyed patients ranging in age from 40 to 74, suggesting a significant risk of reporting and recall bias, particularly for postmenopausal women being asked about their prior menstrual symptoms. Similarly, Stewart surveyed 81 menstruating women between ages 18–55 that self-recorded their migraines and menstrual cycles over 98 days [18]. Stewart noted an increased incidence of migraine perimenstrually—days 0 and 1 had OR of 2.04 (95% CI, 1.49–2.81) and days −1 and −2 had OR of 1.80 (95% CI, 1.40−2.30).

Johannes’ survey studied 28 menstruating women age 22–29 via 4-month self-reported diaries and found that these women reported a higher incidence of migraine during the first 3 days of menses compared to the rest of the cycle (OR 1.66; 95% CI, 1.21–2.26) [15]. Though Johannes’ findings were supported statistically, it was given a low GRADE score because of the study design and risk of reporting bias. In a unique study, Pringsheim surveyed 50 male-to-female transgenders taking antiandrogen and estrogen therapy and had a history of migraine [6]. Pringsheim found that the prevalence of migraines in male-to-female transgenders (26%) was significantly higher than cis-gender males (7.5%) in the population (*p* < 0.05), but was not significantly different from the prevalence of migraines in cis-gender females (25%). These findings suggested that estrogen might be a causative factor in migraine pathogenesis.

### Estrogen’s effects on migraines in postmenopausal women

As menopause is a natural state of estrogen deficiency, postmenopausal women have been studied to investigate the effects of estrogen and the use of HRT on migraines. In 2003, Misakian published a cross-sectional study of 17,107 postmenopausal women and investigated how the use of HRT affects migraines [8]. Misakian found that HRT use significantly increased the risk of experiencing a migraine in postmenopausal women (13% vs 9%, *p* < 0.001). This type of study has been conducted by other researchers as well, yet has yielded varying results. Brandes conducted a comprehensive cross-sectional study of 18,221 postmenopausal women and stratified the results into three groups based on the dose of HRT being taken—low (< 0.3 mg/day), intermediate (0.625 mg/day), and high (> 0.9 mg/day) [5]. Brandes reported that the intermediate dose group had a significantly lower risk of migraine frequency (OR 1.28; 95% CI, 1.10–1.48, *p* = 0.001) than the low (OR 2.00; 95% CI, 1.51–2.65, *p*=0.001) and high (OR 1.72; 95% CI, 1.39–2.13, *p* = 0.002) groups compared to the general population. We considered both of these studies to be high GRADE evidence because of their large sample sizes and efforts to minimize confounding bias; however, it should be noted that both studies utilized patients from the Women’s Health Study and that there is likely a large overlap in the patients used in both studies.

Like Brandes and Misakian, Facchinetti also studied the effects of HRT on migraine pathogenesis in postmenopausal women [11]. Facchinetti conducted a RCT of 38 postmenopausal women and stratified the results into three groups depending on the amount of HRT administered. In contrast to Brandes’ study, which showed a decrease in migraine frequency with 0.625 mg/day estradiol dosing, all three HRT regimens in Facchinetti’s study significantly increased migraine attack frequency (2.2 days per month vs 3.8, *p* < 0.001), (3.4 days per month vs 4.9, *p* < 0.001), (3.4 days per month vs 5.6, *p* < 0.001) over the course of 6 months, and patients also reported increased severity of headache after starting therapy. Notably, two of the three dosing regimens used by Facchinetti were significantly higher than the doses used by Brandes. Although the results between the two studies differed, we still considered the Facchinetti study to be moderate GRADE because it was a RCT with strong statistics that was limited only by small sample size. The conflicting data on HRT in postmenopausal women, especially compared to the estrogen withdrawal hypothesis in menstruating women, highlights the complexity of estrogen’s role in the pathogenesis in migraine headaches. Overall, these findings suggesting that postmenopausal women with a history of migraine are sensitive to increases in estrogen caused by HRT despite being in a low estrogen state.

### Evidence quality/GRADE

Using the GRADE framework, we assessed 3 studies to be high GRADE, 6 as moderate, 6 as low, and 4 as very low. The 3 high GRADE studies were a RCT [7] and two cross-sectional studies [5, 8] which had very large sample sizes and narrow confidence intervals in their statistical analysis. The majority of our included studies were rated as moderate or low mainly due to smaller sample size or the presence of confounding bias. Lastly, the 4 studies rates as very low were survey/diary-based [6, 15, 17, 18] and had a high risk of confounding and recall bias.

## Discussion

The purpose of this study was to provide an updated review of the literature to better characterize the relationship between estrogen and migraines. While previous systematic and literature reviews on hormones and migraines have been conducted, our review focuses specifically on the effects of estrogen in isolation and includes more recent studies compared to prior reviews. The previous systematic review investigating this topic was conducted in 2006 and was impactful [5]; however, our paper advances the topic by including newer studies conducted in the last decade and also by greatly expanding on experiments that utilized hormone replacement therapies as a means for studying estrogen withdrawal. Our investigation also expanded the discussion in the population of post-menopausal women. We believe this provides greater evidence to the link between estrogen and migraines.

During our review, we identified 19 studies that met our inclusion criteria. Of these 19 studies, 12 studied the role of estrogen withdrawal in the precipitation of menstrual migraine. The remainder studied the role of hormone replacement therapy (HRT) in postmenopausal women, the effects of pregnancy, and the effects of fluctuating estrogen level on migraine activity. Overall, we found that many of the included studies fell into one of two categories: estrogen withdrawal migraines or migraines associated with fluctuations in estrogen. In general, nearly all studies concluded that estrogen withdrawal, in particular after a period of priming, significantly increased the likelihood of migraine. Various study designs and approaches have been used to support this theory. We believe that the topic of estrogen withdrawal migraine has been studied sufficiently; however, it became apparent during our review that the pathophysiology of migraines is complex and not fully attributable to estrogen and its withdrawal alone. In particular, many studies focusing on postmenopausal women with migraines found that use of HRT actually increased the incidence of migraine.

As our study primarily focused on only estrogen and not other variables involved in migraines, more general studies were excluded from our final analysis. Further broadened systematic reviews are warranted and would help create a more holistic understanding of migraine pathology. The major limitation in this study was that the majority of studies we screened were confounded by the interplay between menstrual cycles and migraines. While the menstrual migraine is one of the most common subtypes of migraines, the studies we reviewed suggest that the pathogenesis of migraines varies when comparing menstrual migraines, postmenopausal migraines, and non-hormonal migraines. These different subtypes must first be identified and then studied independently to minimizing the effects of confounding variables.

Lastly, a final limitation in our study design was that we focused on clinical research and excluded and studies with data only in the preclinical (animal) stages. Although migraines are extremely common in the population, there are a significant number of preclinical studies that could aid in understanding this disease. Many of the preclinical trials we excluded from our analysis contained insight into the molecular interactions of estrogen with neuromodulators involved in nociception and vascular regulation that are likely to play a role in migraine pathogenesis. A follow-up systematic review that is not constrained to only clinical research would help validate the results of clinical studies that have yet to find an explanation for their findings. In spite of the above limitations, we believe our study highlights the important and not fully understood role that estrogen plays in the pathogenesis of migraines. Further studies are needed to classify different types of migraines, understand various precipitating factors, guide clinical decision making, and reveal new therapeutic targets for medical, surgical, or behavioral intervention.

## Conclusion

The pathophysiology of migraines has proven to be a complex phenomenon. Estrogen is implicated in migraine pathophysiology, but its roles are widespread and still not completely understood. Our scoping review found that most studies on the topic have agreed that the withdrawal of estrogen is a key factor in migraine pathogenesis. This has been extensively studied in both menstruating and postmenopausal women. However, we also found that the pathogenesis of migraines is more complex than can be attributed solely to the withdrawal of estrogen. We believe further study is warranted to differentiate the effects of estrogen on different study populations and also investigate what other hormones, neurotransmitters, and factors play into the pathogenesis of migraine.

## Data Availability

All data generated or analyzed during this study are included in this published article [and its supplementary information files].

## Abbreviations

MRM: Menstrual-related migraines
HRT: Hormone replacement therapy
RCT: Randomized control trial
